# Knowledge and Behavior of Saudi College Athletes toward Energy and Sports Drinks with an Emphasis on Microbial Quality and Safety

**DOI:** 10.3390/sports6030060

**Published:** 2018-07-09

**Authors:** Sulaiman Omar Aljaloud

**Affiliations:** College of Sports Sciences and Physical Activity, King Saud University (KSU), P.O. Box 1949, Riyadh 11441, Saudi Arabia; saljaloud@ksu.edu.sa

**Keywords:** sports drinks, energy drinks, knowledge, behavior, microbial quality, safety, university athletes

## Abstract

The aim of this study was to assess the knowledge and behavior of university athletes in Saudi Arabia regarding energy drinks (EDs) and sports drinks (SDs). In addition, the microbiological quality of available local drinks was also assessed. The presence of total bacterial coliform count, *Escherichia coli*, *Salmonella*, and *Staphylococcus aureus* on these drinks was also determined. Of the 120 university athletes surveyed, 69 were currently using SDs, and 51 were using EDs. The study consisted of a 10-question survey assessing the athletes’ sociodemographic characteristics, personal habits, and SD- and ED-related knowledge and habits. With regard to the question about the primary reason for using EDs, *n* = 26 respondents (50.98%) reported that providing energy (speed, strength, and power) was the main reason for consuming these beverages. Conversely, *n* = 25 respondents (36.23%) believed that recovery from injury or illnesses was the main reason for the consumption of SDs. A majority of university athletes (*n* = 43, 62.32%) who used SDs believed that SDs were safe to use, while *n* = 22 respondents (43.14%) were unaware of any adverse health effects associated with the consumption of EDs. Of the total of 26 SDs and EDs tested, microbial contamination was present in only two products. Microbial levels and the total bacterial count for most of the samples were very low (<1 log CFU/mL). However, some drinks had a slightly higher microbial level, which could be harmful or cause spoilage with improper storage. Thus, our findings suggest that improvements in microbial quality are needed for these beverages.

## 1. Introduction

The consumption of sports drinks (SDs) and energy drinks (EDs) has become a popular practice worldwide, especially among athletes. SDs and EDs are being marketed to athletes and over consumption of these drinks could potentially lead to negative health consequences [1]. SDs and EDs are significantly different products, and the terms should not be used interchangeably. SDs are flavored beverages that usually contain carbohydrates, minerals, and electrolytes and sometimes vitamins or other nutrients while EDs drinks typically contain stimulants, such as caffeine with varying amounts of carbohydrate, protein, amino acids, vitamins, sodium, and other minerals [2]. These drinks represent a large and growing beverage industry. Marketing strategies for SDs include promoting the following benefits: optimization of athletic performance and replacement of fluids and electrolytes lost in sweat during and after exercise, an increase in energy levels, reduction of fatigue, enhanced focus, tolerance for additional training, ability to cope with pain, providing energy (speed, strength, and power), and enhancing mental alertness [3]. SDs are different products than EDs. The former are flavored beverages that contain carbohydrates, minerals (sodium, potassium, calcium, and magnesium), electrolytes, and sometimes vitamins (vitamin B12 and vitamin B6) or other dietary supplements. Although the term “energy” can be perceived to imply calories, EDs typically contain stimulants such as caffeine and taurine, guarana, and varying amounts of carbohydrates, proteins, vitamins, and other minerals [2]. The Saudi government has decided to ban ED advertising altogether. This ban also applies to the distribution of free energy drinks to users of all ages and forbids the sale of energy drinks in eating places such as restaurants and cafeterias, educational and health facilities, and private sports clubs [4]. The worldwide market for EDs is reaching nearly $15 trillion dollars, and the general consumption of EDs has increased accordingly, particularly among youth, adolescents, and university students. The consumption rates among this demographic group are growing because of the concentration-enhancement and fatigue-relieving effects of energy drinks [5,6,7], yet there is relatively little information available about the safety and quality of these products. Commercial SDs such as Powerade, Gatorade, Vitamin water, Sobe Lifewater, Revive, and All Sport have a specifically limited function for athletes and should only be ingested when there is a need for more rapid replacement of electrolytes in combination with water during episodes of prolonged vigorous sports participation and intense physical activity [8,9]. Caffeine is no longer banned by the World Anti-Doping Agency (WADA). However, one would only use caffeine under the guidance of a sports medicine professional or sports dietitian, and individual responses to caffeine should be evaluated with care.

The ED Red Bull is a common carbonated taurine, sugar, and caffeinated beverage that claims to “vitalize the body and mind.” The use of Red Bull as an ED is supported by a fair amount of scientific research and endorsed by recognized sports professionals [10,11,12,13,14]. There are many ways to evaluate the microbiological quality and safety of the EDs available in local stores, and these tests need to be conducted regularly in order to ensure their microbiological quality. However, overdosing can negatively affect reaction time and alertness [8,15,16,17,18].

The consumption rates of EDs and SDs are increasing because of the concentration enhancement and fatigue-relieving effects of these drinks, yet there is relatively little information regarding the safety and quality of these products. To our knowledge, there is very limited published information related to the consumption of EDs and SDs among Saudi university athletes or the microbial quality and safety of these drinks. Therefore, the present study was conducted to explore the attitudes of Saudi college athletes toward EDs and SDs as well as the microbial quality and safety of these beverages and the extent to which the athletes use them.

## 2. Methods

### 2.1. Sampling and Survey Questionnaires

The survey method was discussed with professional athletes in the capital city of Saudi Arabia, Riyadh. The questionnaires were designed to understand knowledge and behavior of Saudi college athletes toward EDs, SDs, and on safety of these drinks. Approval was obtained from research ethics committee. Male athletes (120) randomly selected were recruited from the most popular universities in Riyadh city and those who were willing to participate in the survey were included for the study. Recruited athletes included Saudis as well as foreign nationals.

A consent form was placed on top of the survey for the sports and health department policy on using human subjects along with a description of subject’s specifications and the nature of the survey. The questionnaires were designed to help the researchers understand the athletes’ use and perceptions of SDs and EDs. Two groups of questionnaires were established for this survey, and the results were compared and analyzed to determine the use of EDs and SDs by the athletes. In order to collect data for each of the study’s objectives, we developed a questionnaire consisting of ten questions divided into various categories including reasons for consumption, personal beliefs, knowledge, behavior, and safety. The questions also related to the frequency of SD and ED purchases as well as factors that might be considered by the athletes when purchasing these products.

### 2.2. Data Collection

Approximately three months were required to administer the surveys and collect data from the participants. During the months of January and March 2016, a survey was given to each athlete. The time and location for each survey were arranged and announced at least two weeks in advance. It took approximately 10 min to distribute the surveys and provide instructions, and participants were allowed 25 min to answer the questions.

### 2.3. Sampling for Quality and Safety of Drinks

Twenty-six different commercial SDs and EDs were collected from local stores in Riyadh, Saudi Arabia and randomly labeled with a unique identification number. For example, three digits represented the day of month and time (e.g., 110 meant the first day of the month at 10 a.m.). All products were purchased in duplicate from each store and then shipped to the Laboratory of Food Microbiology at North Carolina A&T State University in Greensboro, North Carolina, USA for microbial quality and product safety analysis.

### 2.4. Microbial Analysis

From each commercial sample, 25 mL were placed in 100 mL sterilized Brain Heart Infusion (BHI) broth and mixed carefully. Samples were then incubated at 37 °C for 4 h to allow for microbial cell recovery, if present. One milliliter of the individual sample was then withdrawn and diluted with 0.1% peptone water. The appropriate dilutions were plated onto identical selective agar plates. To obtain the total bacterial count, samples were plated on BHI agar. To test for coliform bacteria and *Escherichia coli*, samples were plated on Violet Red Bile Glucose (VRBG) agar and MacConkey agar, respectively. Likewise, samples were plated on Xylose Lysine Desoxycholate (XLD) agar for *Salmonella*, and on *Baird-Parker Agar Base* (BPAB) for *Staphylococcus aureus* count.

### 2.5. Statistical Analysis

The Statistical Analysis System (SAS, Inc., Cary, NC, USA) was used to analyze the statistics for this study. The dependent variables were the athletes’ attitudes measured by 10 questions on the questionnaire that were divided into different categories, including reasons for consumption, personal beliefs, knowledge, behavior, and safety. The independent variables were the university athletes’ responses. The results were analyzed using chi-square tests with a significance level set at 0.05.

## 3. Results

Table 1 shows the number of college athletes by age that participated in the survey. Of the 120 participants who responded to the survey, *n* = 11 (9.2%) were 18 years of age or younger, *n* = 96 (80.0%) were 19 to 23 years of age, and *n* = 13 (10.8%) were 24 years of age or older. All 120 athletes were enrolled as students at King Saud University and included freshmen (*n* = 11; 9.2%), sophomores (*n* = 41; 34.2%), juniors (*n* = 26; 21.7%), seniors (*n* = 35; 29.2%), and graduate students (*n* = 7; 5.8%). The survey consisted of 10 questions. The athletes involved in this study consumed both SDs and EDs. Of the 120 athletes surveyed, *n* = 69 (57.50%) were currently taking SDs and *n* = 51 (42.50%) were currently taking EDs (mean age and standard deviation were 21.43 ± 1.77).

Table 2 summarizes the athletes’ responses. In the first question, we asked athletes about their overall impressions and attitudes toward these products. Of the 69 university athletes using SDs, *n* = 41 (59.42%) had a positive impression of SDs, *n* = 18 (26.09%) had a negative impression, and *n* = 10 (14.49%) were neutral about the use of these products. Similarly, of the 51 college athletes using EDs, *n* = 29 (56.86%) thought positively of EDs, *n* = 13 (25.49%) felt negatively about EDs, and *n* = 9 (17.65%) were neutral regarding the use of these beverages. For the second question relating to the frequency of consumption of SDs, the results showed a high percentage of university athletes used SDs daily (*n* = 32; 46.38%), followed by those who consumed SDs weekly (*n* = 20; 28.99%), monthly (*n* = 1; 17.25%), and rarely (*n* = 5; 7.25%). Of the athletes using EDs, *n* = 19 (37.25%) used EDs daily, *n* = 16 (31.37%) used EDs weekly, *n* = 11 (21.57%) used EDs monthly, and *n* = 5 (9.80%) rarely used EDs.

For questions concerning the main reason for using EDs and SDs, the results showed that college athletes use these products for different reasons. For example, of the athletes who use EDs, *n* = 26 (50.98%) believed that an energy boost (speed, strength, and power) was the main reason for using EDs, followed by *n* = 13 (25.49%) for recovery from an injury or illness, *n* = 6 (11.76%) to enhance tolerance for additional training, and *n* = 2 (3.92%) each to enhance the ability to cope with pain and improve endurance. However, of the athletes using SDs, *n* = 25 (36.23%) reported using SDs to recover from an injury or illness, followed by *n* = 18 (26.09%) to provide energy (speed, strength, power), *n* = 9 (13.04%) to enhance the ability to cope with pain, *n* = 8 (11.59%) to improve endurance, and *n* = 5 (7.25%) to enhance tolerance for additional training.

A majority of college athletes who consumed SDs (*n* = 27; 39.13%) thought SDs are used as a dietary supplement, followed by *n* = 22 (31.88%) who had no information, *n* = 19 (27.54%) thought SDs were used to boost energy, and *n* = 1 (1.45%) felt that SDs acted as a stimulant. Similarly, for the athletes who used EDs, *n* = 23 (45.10%) thought EDs boosted energy, *n* = 14 (27.45%) did not have any information, *n* = 12 (23.53%) thought EDs were a dietary supplement, and only *n* = 2 (3.92%) considered EDs as stimulants.

Of the athletes using SDs, most (*n* = 19; 27.54%) received information on SDs from family members or friends, followed by *n* = 16 (23.19%) athletes who learned about them from retail stores. However, fewer than 10% reported their sources of information to be a nutritionist or dietician, an online website, or another source. Furthermore, most athletes using EDs (*n* = 21; 41.18%) received information about EDs from family members or friends; however, fewer than 10% reported their sources of information on EDs to be from a retail store, a nutritionist or dietician, online websites, a coach or physician, or another source.

Table 3 lists athletes’ responses to Questions 6–8 regarding their perceptions of the safety of drinks. Of the 69 college athletes using SDs, *n* = 38 (55.07%) agreed that SDs had no associated side effects, whereas *n* = 22 (31.88%) did not know about the products’ side effects, and *n* = 9 (13.04%) believed that there were no side effects associated with these products. Conversely, a majority of the athletes using EDs (*n* = 21; 41.18%) agreed that EDs had no associated side effects, and *n* = 17 (33.33%) thought these products were safe to consume. Only *n* = 13 (25.49%) of the 51 college athletes who used EDs had no information about these products.

A majority of college athletes who used SDs (*n* = 41, 59.42%) agreed that these beverages had no effect on the immune system, whereas *n* = 13 athletes (18.84%) believed that SDs did have an effect on the immune system. Overall, of the 69 athletes who used SDs, *n* = 15 (21.74%) did not have any information related to the effects of SDs on their immune system. Conversely, a majority of athletes who used EDs (*n* = 22; 43.14%) thought EDs had no effect on the immune system, followed by *n* = 17 (33.33%) who had no information about the potential risk of EDs on the immune system. A large number of the study participants who used SDs (*n* = 43; 62.32%) agreed that SDs were safe to consume, whereas *n* = 15 (21.74%) had no indication about product safety. Of the 69 athletes who used SDs, *n* = 11 (15.94%) believed SDs were not safe to consume. Conversely, *n* = 22 athletes (43.14%) who used EDs had no information regarding the safety of EDs, followed by *n* = 18 (35.29%) who thought that these products were safe to consume. Only *n* = 11 (21.57%) of the 51 athletes who used EDs believed that these beverages were unsafe to consume.

Table 4 lists the SDs that the study participants reported using. We were interested in knowing more about the types of SD products that were being used among these athletes. The participants reported using more than seven different products most frequently. Our results showed that Gatorade consumption (*n* = 34; 49.28%) was the highest among all these drinks followed by Powerade, Sobe Lifewater, All Sport, Vitamin water, and Revive. Consumption of SDs not listed in Table 4 was reported by *n* = 11 athletes (15.94%).

Table 5 lists the EDs that our college athletes reported consuming during the session covered by this survey questionnaire. We were interested in identifying the type of energy drinks that were being used. University athletes reported using over 12 different products. Our results showed that Red Bull (*n* = 20, 39.22%) and Code Red (*n* = 13; 25.49%) were the most popular EDs, followed by Bugzy (*n* = 4; 7.84%), Vault (*n* = 3; 5.88%), and Power Horse, Black, Boom Boom, and AMP (*n* = 2; 3.92% each).

Table 6 lists the 26 different commercial beverages by an assigned product number, including 20 EDs and six SDs (acquired from local stores in Riyadh) and their respective caffeine levels, pH values, and total bacterial counts. The laboratory testing results showed that none of the commercial samples contained harmful pathogens. The results also indicated an absence of *Escherichia coli*, *Salmonella species*, and *Staphylococcus aureus*. The total plate count of bacteria for most of the samples was very low (<1 log CFU/mL). Only two tested samples had a bacterial population of approximately 2.00 Log CFU/mL. These samples had low caffeine concentrations and higher pH values compared to other samples. In addition, the microbial populations in six different sports drinks were below a detectable level.

## 4. Discussion

In this study, we conducted a survey concerning the consumption of SDs and EDs among college athletes (*n* = 120) with an emphasis on the evaluation of the microbial quality and safety of these products. Based on these results, the participating athletes in our study generally appeared to have a lack of knowledge about popular SDs and EDs. Because college athletes are typically under strong pressure to perform well, some are always in need of SDs and EDs to enhance performance. Limited data regarding the consumption of SDs and EDs has been published in Saudi Arabia; thus, there is an inherent need to improve the understanding of SD and ED use among athletes. Each year, new SD and ED products become available in the growing Saudi Arabian market, and intake of these beverages among college athletes has also been increasing [19]. Some research studies in the past have shown a positive correlation between the nutrition knowledge of college students and the quality of their energy drink intake [20]. This finding is similar to other studies conducted on the SD and ED consumption patterns of college students in the United States and in the Philippines [20,21,22].

Of the 120 college athletes surveyed about SD and ED consumption, we found that *n* = 69 (57.50%) were currently taking SDs and *n* = 51 (42.50%) were currently taking EDs. The survey results showed that a majority of athletes surveyed use these products throughout the year to either improve or enhance performance or only to improve their health. In an earlier study [20], the authors noted that EDs among college students were primarily used to improve and enhance performance. Likewise, in a study involving Saudi college students, 73% of the students reported using energy drinks [1,3,4].

In the present study, the results showed that of the 69 college athletes who used SDs, *n* = 32 (46.38%) consume them daily, whereas of the 59 athletes who used EDs, *n* = 19 (37.25%) did so on a daily basis. Trunzo et al. [22] also reported that college students consumed EDs at least 2–5 days per week. Similarly, our results also showed that 40% of the students consumed EDs at least once a week and 34% were likely to increase the frequency of intake to twice a week. Conversely, professional athletes reported that the frequency of drinking EDs was at least once per week (36%) with the likeliness of increased intake to more than three times per week [20].Similarly, in another study, it was noted that 18.8% of young adults consumed energy drinks at least weekly [23]. In addition, the average (mean ± standard deviation) number of cans or bottles of EDs consumed among nursing students per week after studying for exams was reported to be 1.63 ± 2.64, with a range of 1–30 cans or bottles consumed [24].

Regarding the primary reason for using EDs and SDs, results from the current study showed that college athletes use these products for different reasons. For example, 26 athletes (50.98%) using EDs reported gaining speed, strength, and power as the main reason for using EDs. However, of the athletes using SDs, *n* = 25 (36.23%) reported using SDs to recover from an injury or illnesses. Similarly, in the study by Reid et al. [25], EDs were used most commonly to enhance energy (50%), combat sleepiness (45%), improve and enhance academic performance (40%), and enhance performance during sports (23%). In another study by Bawazeer and AlSobahi [26], the main reason for ED consumption among college students was to study for exams or to finish a project (31.4%).

Our results showed that of the 69 college athletes who used SDs, the majority (*n* = 43; 62.32%) agreed that SDs were safe to consume, *n* = 15 (21.74%) had no information about their safety, and *n* = 11 (15.94%) did not consider SDs safe to use. Conversely, a majority of the 51 athletes using EDs (*n* = 22; 43.14%) had no information regarding the safety of EDs, followed by *n* = 18 (35.29%) of athletes who believed that these products were safe to consume. Only *n* = 11 of the 51 athletes using EDs (21.57%) felt that these products were not safe. In a previous study, Bawazeer and AlSobahi [26] found the most important reason for using EDs was to increase energy levels in general (32.8%) while other reasons included counteracting sleep deprivation (12.8%) [27], imitating friends (11.4%), or being more alert while driving (8.5%). Another study reported that the main reason that college students consumed EDs was to work overtime to finish a course project [20]. In another study, taste-seeking athletes (31%) endorsed pleasurable taste, energy-seeking athletes (24%) endorsed function and taste motives, and hedonistic athletes (33%) endorsed pleasure and sensation-seeking motives [28]. In a study by Kim and Kim [24], additional reasons for energy drink consumption included enhanced recovery (79.9%), concentration (29.3%), and curiosity (22.0%) among university nursing students.

A study by Emond, Sargent, and Gilbert-Diamond [29] showed that athletes received most of their information about energy drinks from stores, college students, friends, and manufacturers that advertise primarily on television and social media. These results were similar to our study’s results in which most college athletes who use SDs (*n* = 19; 27.54%) reported a family member or friend as their main source of information about these products, followed by retail stores (*n* = 16; 23.19%), a coach or physician (*n* = 14; 20.29%), a nutritionist or dietician (*n* = 8; 11.59%), and online websites (*n* = 7; 10.14%). In addition, a large number of athletes using EDs (*n* = 21; 41.18%) reported a family member or friend as their primary source of information about these products. However, fewer than 10% of the athletes using EDs reported their sources of information to be a nutritionist or dietician, websites, or other sources.

In the present study, college athletes using EDs reported using over 12 different products most frequently. Our results showed that Red Bull (*n* = 20; 39.22%) and Code Red (*n* = 13; 25.49%) were the most popular EDs, followed by Bugzy (*n* = 4; 7.84%), Vault (*n* = 3; 5.88%), AMP, Bison, Black, Boom Boom, and Power Horse *n* = (2; 3.92%). Similarly, Capule et al. [20] also reported Cobra, Red Bull, and Monster Energy as the most popular products used by students. These authors reported that among the most popular brands of drinks, Cobra was significantly more popular among professionals while Red Bull and Monster were more popular among students.

Quality assurance and standardization are two important key factors in the food products development. Medicinal plant materials carry a great number of bacteria and molds often originating in soil, while a large range of bacteria and fungi occur naturally on the surface of herbs. Harvesting, handling and production may also cause additional contamination and microbial growth in such source of plant ingredients. Selection of plant materials based on quality, standardization, methods of preparation, and enforcement of regulation regarding appropriate labels are measures which will improve the quality and acceptance of herbal preparations as therapeutic agents [30]. Okunlola et al. [31] studied the microbial quality of different medicinal products in Southwestern Nigeria which included 21 different brands of herbal medicine and found that the tested products contained several harmful microorganisms including *E. coli*, *Salmonella* spp., *Staphylococcus aureus*; and fungi. Similarly, significant contamination with bacteria and fungi was reported during the investigation of the microbial quality of herbal medicines collected from the shops in the Nelson Mandela Metropolis. The presence of *Salmonella* and *E. coli* in herbal powder and tablets have been also reported earlier [32]. Therefore, these results along with our findings emphasize for the need to improve the quality of products used by athletes.

## 5. Limitations and Strengths

Since this survey only included Saudi college athletes, it may not be representative of college athletes worldwide. Furthermore, availability of EDs and SDs varied from region to region limits the choice of samples type and size needed to represent the large population. We understand that this is a small representation of the tested products. Nonetheless, the present investigation on the factors that affect EDs and SDs consumption among student athletes is significant because it reveals a pressing need for the establishment of regulations and guidelines for the marketing of EDs and SDs as well as requirements for accurate disclosure of their potential side effects. Consequently, there have been recommendations that the sale and use of EDs be restricted in universities, schools, and even coffee shops [33].

## 6. Conclusions and Future Directions

Many college athletes consume SDs and EDs as a part of their daily routines. These products are often consumed without the athletes’ having a full understanding of the potential benefits, negative effects, and associated risks because of a lack of consultation with sports nutrition professionals.

The findings of this study will help us to further expand our knowledge on the use of SDs and EDs. Based on these results, we can develop an education program to educate them about the benefits as well as possible negative effects of these drinks. However, more research and increased public awareness are thus needed to bring about an improvement in the education of athletes with regard to SDs and EDs. This education must highlight the differences between SDs and EDs and their associated potential health risks. In this study, we also evaluated the microbiological quality and safety of 26 of the most common EDs and SDs sold in local stores in Saudi Arabia. The samples were selected based on availability and affordability. Our results confirmed the safety and quality of all tested products as they showed low levels of contamination by total bacterial population and the absence of any pathogenic bacteria.

Regarding the consumption of SDs and EDs by athletes, we should:

- Improve the education of athlete’s regarding SDs and EDs. This education must highlight the differences between SDs and EDs and their associated potential health risks.
- Be aware that these products could pose potential health risks primarily because of their stimulant content; therefore, they are not appropriate for athletes, and their consumption should be avoided.
- Counsel athletes whose routine consumption of carbohydrate-containing SDs should be avoided or restricted.
- Educate athletes regarding the specifically limited function of SDs. These drinks should only be ingested when there is a need for more rapid replenishment of carbohydrates in combination with water during sports participation or other intense physical activity.
- Promote water, not SDs or EDs, as the principal source of hydration for athletes.
- Conduct additional research related to the microbial content and quality of not only SDs and EDs but also other popular sports supplements that are currently being sold in Saudi markets.

## Figures and Tables

**Table 1:** Participant demographics (*n* = 120).

Characteristic	*n* (%)
Age	
18 or younger	11 (9.2)
19 to 23	96 (80.0)
24 or older	13 (10.8)
Class standing	
Freshman	11 (9.2)
Sophomore	41 (34.2)
Junior	26 (21.7)
Senior	35 (29.2)
Graduate	7 (5.8)
SD and ED consumption	
Using SDs	69 (57.50)
Using EDs	51 (42.50)

Note: The results showed that the responses differed significantly (*p* < 0.0001) from the hypothesized value (0.05) indicating that the demographic data was limited to age and class standing. SD = sports drink; ED = energy drink.

**Table 2:** Knowledge and attitudes towards safety drinks (*n* = 69) and energy drinks (*n* = 51) among college athletes.

Responses	SDs *n* (%)	EDs *n* (%)
1. What is your overall impression and attitude toward these products?		
Positive	41 (59.42)	29 (56.86)
Negative	18 (26.09)	13 (25.49)
Neutral	10 (14.49)	9 (17.65)
2. How often do you consume these products?		
Every day	32 (46.38)	19 (37.25)
Once per week	20 (28.99)	16 (31.37)
Once per month	12 (17.25)	11 (21.57)
Rarely	5 (7.25)	5 (9.80)
3. What is the main reason for consuming these products? (Most important reason)		
Provide energy (speed, strength, power)	18 (26.09)	26 (50.98)
Recover from an injury or illness	25 (36.23)	13 (25.49)
Improve endurance	8 (11.59)	2 (3.92)
Enhance tolerance for additional training	5 (7.25)	6 (11.76)
Enhance ability to cope with pain	9 (13.04)	2 (3.92)
Other	4 (5.80)	2 (3.92)
4. What are these products used for?		
Elements to boost energy	19 (27.54)	23 (45.10)
As a dietary supplement	27 (39.13)	12 (23.53)
As stimulants	1 (1.45)	2 (3.92)
Do not know	22 (31.88)	14 (27.45)
5. How do you obtain information about these products?		
Coach or physician	14 (20.29)	4 (7.84)
Nutritionist or dietician	8 (11.59)	5 (9.80)
Family or friends	19 (27.54)	21 (41.18)
Online	7 (10.14)	4 (7.84)
Retail store	16 (23.19)	8 (15.69)
Others	5 (7.25)	9 (17.65)

Note: The results showed that the response does differ significantly (*p* < 0.0001) from the hypothesized value (0.05) indicating knowledge and attitudes towards SDs and EDs differ for each individual. SD = sports drink; ED = energy drink.

**Table 3:** Safety of sports drinks (*n* = 69) and energy drinks (*n* = 51) among college athletes.

Response	SDs *n* (%)	Eds *n* (%)
6. Are you able to identify if there are any side effects of these products?		
Yes	9 (13.04)	17 (33.33)
No	38 (55.07)	21 (41.18)
Do not know	22 (31.88)	13 (25.49)
7. Should you worry about the effects of these products’ on your immune system?		
Yes	13 (18.84)	12 (23.53)
No	41 (59.42)	22 (43.14)
Do not know	15 (21.74)	17 (33.33)
8. Do you consider these products safe to use?		
Yes	43 (62.32)	18 (35.29)
No	11 (15.94)	11 (21.57)
Do not know	15 (21.74)	22 (43.14)

Note: The results show that the response for the “safe to use these products” question differs significantly (*p* < 0.0001) from the hypothesized value (0.05) indicating that the responses differ for each study participant. SD = sports drink; ED = energy drink.

**Table 4:** Type of sports drink and frequency of use among university athletes (*n* = 69).

Brand of Sports Drink	*n*	%
9. Which of these sports drinks have you used most frequently?		
Powerade	7	10.14
Gatorade	34	49.28
Vitamin water	4	5.80
Sobe Lifewater	6	8.70
Revive	3	4.35
All Sport	4	5.80
Other	11	15.94

Note: The results show a *p*-value of 0.0001; thus, we can conclude that there is a statistically significant difference between the type of sports drink and frequency of use among college athletes.

**Table 5:** Type of energy drink and frequency of use among college athletes (*n* = 51).

Brand of Energy Drink	*n*	%
10. Which of these products have you used most frequently?		
Red Bull	20	39.22
Code Red	13	25.49
Bison	2	3.92
Bugzy	4	7.84
Power Horse	2	3.92
Vault	3	5.88
Blu Day	1	1.96
Black	2	3.92
Boom Boom	2	3.92
Shark	0	0.00
AMP (simply Amp)	2	3.92
Other	0	0.00

Note: The results show a *p*-value 0.0001, so we can conclude that there is a statistically significant difference between the type of energy drink and frequency of use among college athletes.

**Table 6:** Quantity of caffeine ^1^, total bacterial count ^2^, and pH values of beverages (EDs and SDs) available in local market.

Product Number	Caffeine (mg/L)	Total Bacterial Count (Log CFU/mL)	pH Value
1	3500	1.60	3.85
2	300	<1.0	4.10
3	280	<1.0	3.00
4	530	1.20	86.00
5	300	<1.0	3.90
6	900	<1.0	4.10
7	320	1.70	3.54
8	210	<1.0	4.15
9	450	<1.0	4.23
10	500	<1.0	3.90
11	120	2.10	3.79
12	310	1.30	3.75
13	500	<1.0	3.80
14	310	1.75	4.05
15	400	<1.0	3.76
16	500	<1.0	3.95
17	160	2.30	4.20
18	50	2.5	4.10
19	10	2.1	3.87
20	290	<1.0	2.70
21	0	<1.0	2.70
22	0	<1.0	4.00
23	0	<1.0	5.60
24	0	<1.0	3.60
25	0	<1.0	5.40
26	0	<1.0	3.75

^1^ For comparison, cola has 100 mg/L and coffee ~ 250 mg/L caffeine; ^2^ <1.0 (CFU/mL) means below detectable levels.
